# Association between triglyceride-glucose index and phenotypic age acceleration: a cross-sectional study based on NHANES database

**DOI:** 10.3389/fphys.2025.1548690

**Published:** 2025-05-01

**Authors:** Zhili Zhao, Yan Liang

**Affiliations:** ^1^ Department of Critical Care Medicine, West China Hospital, Sichuan University, Chengdu, China; ^2^ West China School of Nursing, Sichuan University, Chengdu, China; ^3^ Department of Neurology, West China Hospital, Sichuan University, Chengdu, China

**Keywords:** TyG index, phenotypic age acceleration, insulin resistance, metabolic dysfunction, cross-sectional study, national health and nutrition examination survey (NHANES)

## Abstract

**Objective:**

To investigate the association between triglyceride-glucose (TyG) index and phenotypic age acceleration (PhenoAgeAccel), given the emerging importance of biological aging as a health determinant and the role of insulin resistance in aging-related processes.

**Methods:**

This cross-sectional study analyzed data from 13,291 adults aged ≥20 years in the National Health and Nutrition Examination Survey (1999–2010). The TyG index served as the exposure variable, calculated from fasting triglycerides and glucose levels. PhenoAgeAccel, derived from clinical biomarkers, was the outcome variable. Analyses adjusted for demographic, socioeconomic, and health-related covariates.

**Results:**

A significant non-linear relationship was observed between TyG index and PhenoAgeAccel, with an inflection point at 9.60. In the fully adjusted model, each unit increase in TyG index was associated with 2.21 years increase in PhenoAgeAccel (95% CI: 1.99, 2.43). The association was stronger above the inflection point (β = 8.21, 95% CI: 7.59, 8.82) compared to below it (β = 0.56, 95% CI: 0.29, 0.83).

**Conclusion:**

Higher TyG index levels are significantly associated with accelerated biological aging, particularly above a threshold of 9.60. These findings suggest the importance of metabolic health in biological aging processes and potential interventional strategies.

## 1 Introduction

Biological aging, distinct from chronological aging, is increasingly recognized as a significant determinant of health outcomes (Zane et al., 2024). It reflects the cumulative burden of molecular and cellular damage, influencing morbidity and mortality risks (Lu et al., 2012). Phenotypic age acceleration (PhenoAgeAccel), a biomarker that captures deviations between biological and chronological age, has been validated as a predictor of disease and premature death (Li et al., 2024) The identification of metabolic factors associated with biological aging has become a critical area of research. Insulin resistance (IR), characterized by reduced glucose uptake and lipid metabolism, is one such factor linked to systemic inflammation and oxidative stress, both of which accelerate biological aging (Tucker, 2022). The triglyceride-glucose (TyG) index, a composite marker of IR derived from fasting glucose and triglyceride levels, has gained prominence due to its simplicity and strong association with metabolic disorders and aging-related diseases, such as diabetes and cardiovascular disease (CVD) (Tian et al., 2021; Zhang and Zeng, 2023).

The interplay between metabolic dysfunction and biological aging is influenced by factors such as obesity, inflammation, and molecular changes. For example, visceral adiposity, a major contributor to IR, is associated with PhenoAgeAccel and systemic inflammation (Xu et al., 2024; Huang et al., 2024). These processes are closely tied to oxidative stress, which damages cellular structures and accelerates the aging process (Tucker, 2022). Additionally, molecular markers such as Klotho protein, a regulator of insulin signaling and anti-aging mechanisms, have shown inverse relationships with metabolic dysfunction and aging indicators (Bian et al., 2015). The TyG index has been linked to lower levels of Klotho protein, particularly in populations with metabolic disorders, reinforcing its role as a key mediator in aging-related pathways (Qiu et al., 2024; Lai and Chen, 2024).

Despite the growing evidence of the relationship between metabolic dysfunction and biological aging, few studies have specifically examined the association between TyG index and PhenoAgeAccel. Previous research has identified non-linear relationships and subgroup-specific differences in the effects of TyG index on health outcomes (Zhang and Zeng, 2023; Lin et al., 2024). This study aims to investigate the association between TyG index and PhenoAgeAccel using data from the National Health and Nutrition Examination Survey (NHANES). By examining threshold effects and stratified relationships, this research seeks to provide new insights into the metabolic drivers of biological aging and inform potential intervention strategies for mitigating age-related health risks.

## 2 Methods

### 2.1 Study population

Study Population This cross-sectional study utilized data from the NHANES collected between 1999 and 2010 in the United States. NHANES employs a multistage, stratified sampling method to ensure a nationally representative sample of the U.S. population. Data are collected via standardized questionnaires, physical examinations, and laboratory tests conducted by trained personnel, ensuring high quality and consistency. The study population included 13,291 participants aged 20 years or older. Eligibility criteria required participants to be non-pregnant and to have complete data on both the TyG index and PhenoAgeAccel. Participants with missing data for any critical variables were excluded.

### 2.2 Exposure variable

The primary exposure variable, the TyG index, was calculated using the formula: TyG index = ln[triglycerides (mg/dL) × fasting glucose (mg/dL)/2] (Zhang and Zeng, 2023).

Triglyceride and glucose levels were measured using NHANES-certified laboratory techniques, including enzymatic methods for triglycerides and a hexokinase assay for glucose.

### 2.3 Outcome variable

Phenotypic age (PhenoAge) was calculated using the clinical biomarkers identified in Levine et al., which were selected based on their ability to predict mortality. These include albumin (liver function), creatinine (kidney function), glucose (metabolic function), C-reactive protein (inflammation), lymphocyte percentage (immune function), mean red cell volume (hematological health), red cell distribution width (hematological variability), alkaline phosphatase (liver and bone health), white blood cell count (immune response), and chronological age. The coefficients used for each biomarker were derived from a Gompertz proportional hazard model applied to the NHANES III dataset, as detailed in Levine et al. (2018).

PhenoAgeAccel was then calculated as the residual of PhenoAge after regressing it on chronological age. This measure represents the extent to which an individual’s biological age deviates from their chronological age, with positive values indicating accelerated aging and negative values suggesting decelerated aging.

### 2.4 Covariates

Covariates included the following variables: age (continuous, in years), sex (male or female), race/ethnicity (Non-Hispanic White, Non-Hispanic Black, Mexican American, and Other races including multiracial), poverty-income ratio (PIR) (categorized as low [<1.3], medium [1.3–3.5], and high [>3.5]), education level (less than high school, high school graduate, and more than high school), BMI (continuous, in kg/m^2^), smoking status (never, former, current), drinking status (never, former, mild, moderate, and heavy drinking, as categorized in NHANES), glucose metabolism state (normoglycemia, prediabetes, and diabetes based on fasting glucose and HbA1c levels), hypertension (yes: systolic blood pressure ≥140 mmHg, diastolic blood pressure ≥90 mmHg, or antihypertensive medication use; no: does not meet these criteria), hyperlipidemia (yes: total cholesterol ≥240 mg/dL or lipid-lowering therapy; no: does not meet these criteria), physical activity (METs/week) (categorized as low [<600], moderate [600–1,199], and vigorous [≥1,200]), uric acid (continuous, mg/dL), eGFR (continuous, in mL/min/1.73 m^2^, calculated using the CKD-EPI equation) (Levey et al., 2009), CVD (yes: self-reported history of coronary heart disease, congestive heart failure, heart attack, stroke, or angina; no: does not meet these criteria), and dietary quality measured by the Healthy Eating Index-2015 (HEI-2015) (scored 0–100, with higher scores indicating better diet quality) (Krebs-Smith et al., 2018). Covariates were selected based on their potential confounding effects on the relationship between TyG index and PhenoAgeAccel (Franzago et al., 2022).

### 2.5 Statistical analysis

Continuous variables were expressed as mean ± standard deviation for normally distributed data or median (min, max) for non-normally distributed data, while categorical variables were presented as frequencies and percentages. Group comparisons were performed using χ^2^ tests for categorical variables, Student’s t-tests for normally distributed variables, or Mann-Whitney U tests for non-normally distributed variables. Missing data were handled using multiple imputation via chained equations (MICE) to generate five datasets, reducing potential bias and information loss caused by missing values (Toutenburg and Rubin, 1990; Sterne et al., 2009).

Prior to regression analyses, model assumptions were thoroughly evaluated. Predictor variables were checked for near-zero variance. Multicollinearity was assessed using variance inflation factor (VIF) analysis through stepwise selection. For continuous variables, linearity assumptions were tested using multivariable fractional polynomial (MFP) analysis, which identified appropriate transformations when necessary to ensure linear relationships with the outcome variable.

The relationship between the TyG index and PhenoAgeAccel was analyzed using linear regression models, including both univariate and multivariate approaches. Three models were constructed to explore the association under different levels of adjustment. Model 1 did not include any covariates; Model 2 adjusted for age, sex, and race/ethnicity; and Model 3 further adjusted for additional covariates, including PIR, education level, BMI, smoking, drinking, glucose metabolism state, hypertension, hyperlipidemia, METs/week, uric acid, eGFR, CVD (including coronary heart disease, congestive heart failure, heart attack, stroke, and angina), and HEI-2015. These models were built to assess how the effect size of the TyG index varied with different adjustment strategies, ensuring the robustness of the findings. Model fit was assessed using multiple criteria, including R^2^, adjusted R^2^, AIC, and log-likelihood values. Residual analysis was performed to confirm the validity of model assumptions.

To investigate potential nonlinearity in the association between the TyG index and PhenoAgeAccel, generalized additive models (GAM) and smooth curve fitting were applied (Hastie and Tibshirani, 1987). If nonlinearity was identified, a recursive algorithm was used to determine the inflection point, followed by constructing a two-piecewise linear regression model for each side of the inflection point. The model that best described the association was selected based on log-likelihood ratio tests comparing the two-piecewise regression model with the standard linear regression model.

Subgroup analyses were conducted to explore potential effect modifications. Stratified analyses were performed using linear regression models, stratified linear regression models, and GAM. Continuous subgroup variables were converted into categorical variables based on clinical cut points or tertiles before performing interaction tests.

All statistical analyses were conducted using R software (http://www.R-project.org, The R Foundation). A two-sided P-value <0.05 was considered statistically significant.

## 3 Results

### 3.1 Study sample selection

**FIGURE 1:**
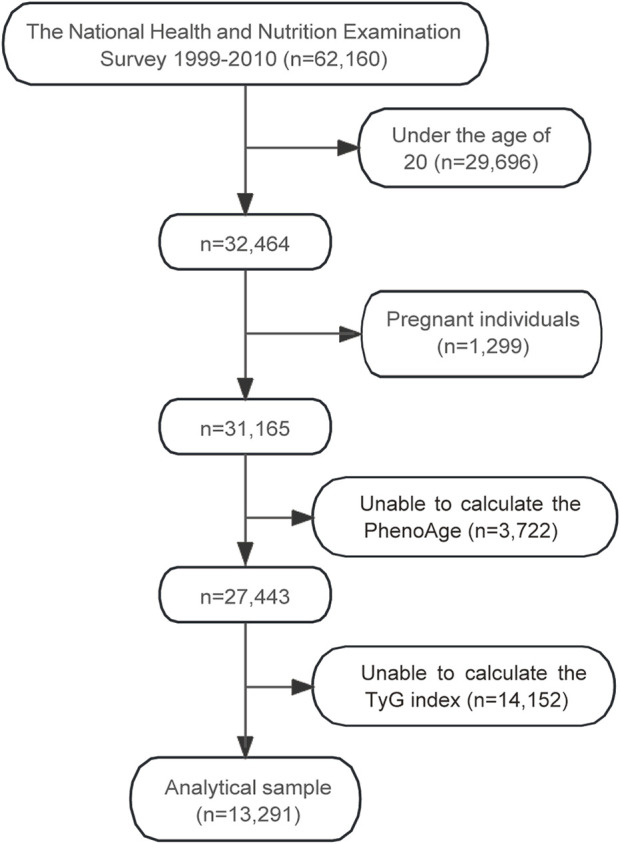
Flowchart of participant selection for the study. Abbreviations: PhenoAge, phenotypic age; TyG, triglyceride-glucose.

The flowchart in Figure 1 illustrates the selection process of participants from the NHANES 1999–2010 dataset, which initially included 62,160 individuals. After excluding participants under the age of 20 (n = 29,696) and pregnant individuals (n = 1,299), 31,165 participants remained. Subsequently, those with insufficient data to calculate PhenoAge (n = 3,722) were excluded, leaving 27,443 participants. Further exclusion of individuals without sufficient data to calculate the TyG index (n = 14,152) resulted in a final analytical sample of 13,291 participants.

### 3.2 Baseline demographic characteristics

The baseline characteristics of participants across TyG index quartiles demonstrated significant differences in demographic, socioeconomic, and lifestyle factors (Table 1). Participants in higher TyG quartiles were generally older, with the mean age increasing from 42.98 years in Q1 to 55.66 years in Q4 (P < 0.05). Gender distribution also varied significantly, with males accounting for a progressively larger proportion in higher quartiles, rising from 41.20% in Q1 to 56.76% in Q4 (P < 0.05). Differences in race and ethnicity were evident, as the proportion of Mexican Americans increased from 14.32% in Q1 to 26.57% in Q4, while non-Hispanic Black participants showed a significant decline from 29.91% in Q1 to 11.71% in Q4 (P < 0.05). Socioeconomic indicators such as PIR and education level exhibited significant gradients, with participants in Q4 more likely to have lower incomes (33.19% low PIR in Q4 vs. 27.14% in Q1) and lower educational attainment (38.37% of Q4 participants had less than high school education vs. 23.62% in Q1, P < 0.05). Smoking status and alcohol consumption also varied, as former smokers and heavy drinkers were more prevalent in Q4 compared to Q1 (P < 0.05).

**TABLE 1:** Basic characteristics of the study participants.

Variables	TyG index quartiles				P-value
	Q1 (6.66–8.27) n = 3,323	Q2 (8.27–8.67) n = 3,322	Q3 (8.67–9.11) n = 3,323	Q4 (9.11–12.48) n = 3,323	
Age (years)	42.98 ± 17.53	50.48 ± 18.53	53.70 ± 18.03	55.66 ± 16.40	<0.05
Gender (n, %)					<0.05
Male	1,369 (41.20%)	1,647 (49.58%)	1766 (53.14%)	1886 (56.76%)	
Female	1954 (58.80%)	1,675 (50.42%)	1,557 (46.86%)	1,437 (43.24%)	
Race/ethnicity (n, %)					<0.05
Non-Hispanic White	1,518 (45.68%)	1,664 (50.09%)	1735 (52.21%)	1,691 (50.89%)	
Non-Hispanic Black	994 (29.91%)	684 (20.59%)	458 (13.78%)	389 (11.71%)	
Mexican American	476 (14.32%)	625 (18.81%)	726 (21.85%)	883 (26.57%)	
Others	335 (10.08%)	349 (10.51%)	404 (12.16%)	360 (10.83%)	
PIR (n, %)					<0.05
Low	902 (27.14%)	930 (28.00%)	949 (28.56%)	1,103 (33.19%)	
Medium	1,252 (37.68%)	1,291 (38.86%)	1,352 (40.69%)	1,326 (39.90%)	
High	1,169 (35.18%)	1,101 (33.14%)	1,022 (30.76%)	894 (26.90%)	
Education level (n, %)					<0.05
Less than high school	785 (23.62%)	967 (29.11%)	1,091 (32.83%)	1,275 (38.37%)	
High school graduate	691 (20.79%)	825 (24.83%)	777 (23.38%)	835 (25.13%)	
More than high school	1847 (55.58%)	1,530 (46.06%)	1,455 (43.79%)	1,213 (36.50%)	
Smoking (n, %)					<0.05
Never	1987 (59.80%)	1734 (52.20%)	1,640 (49.35%)	1,485 (44.69%)	
Former	659 (19.83%)	822 (24.74%)	955 (28.74%)	1,080 (32.50%)	
Now	677 (20.37%)	766 (23.06%)	728 (21.91%)	758 (22.81%)	
Drinking (n, %)					<0.05
Never	474 (14.26%)	424 (12.76%)	478 (14.38%)	499 (15.02%)	
Former	527 (15.86%)	657 (19.78%)	738 (22.21%)	852 (25.64%)	
Mild	1,101 (33.13%)	1,070 (32.21%)	1,098 (33.04%)	973 (29.28%)	
Moderate	607 (18.27%)	480 (14.45%)	366 (11.01%)	354 (10.65%)	
Heavy	614 (18.48%)	691 (20.80%)	643 (19.35%)	645 (19.41%)	
METs/week (n, %)					0.22
Low	1,468 (44.18%)	1,502 (45.21%)	1,420 (42.73%)	1,509 (45.41%)	
Moderate	115 (3.46%)	117 (3.52%)	102 (3.07%)	111 (3.34%)	
Vigorous	1740 (52.36%)	1703 (51.26%)	1801 (54.20%)	1703 (51.25%)	
BMI (kg/m^2^)	26.21 ± 6.03	28.09 ± 6.29	29.56 ± 6.59	30.80 ± 6.17	<0.05
Height (cm)	167.74 ± 9.76	167.91 ± 10.19	167.42 ± 10.38	167.75 ± 10.49	0.26
SBP (mmHg)	117.98 ± 18.15	123.67 ± 19.28	126.34 ± 19.75	129.76 ± 20.03	<0.05
DBP (mmHg)	68.41 ± 11.56	69.56 ± 11.90	70.67 ± 12.26	71.71 ± 12.80	<0.05
Uric acid (mg/dL)	4.94 ± 1.31	5.41 ± 1.35	5.75 ± 1.40	5.97 ± 1.49	<0.05
eGFR (ml/min/1.73 m^2^)	101.11 ± 22.55	93.02 ± 22.66	89.30 ± 23.44	87.56 ± 23.66	<0.05
Glucose metabolism state (n, %)					<0.05
Normoglycemia	2,971 (89.41%)	2,616 (78.75%)	2,134 (64.22%)	1,382 (41.59%)	
Prediabetes	209 (6.29%)	400 (12.04%)	622 (18.72%)	588 (17.69%)	
Diabetes	143 (4.30%)	306 (9.21%)	567 (17.06%)	1,353 (40.72%)	
Hypertension (n, %)					<0.05
No	2,457 (73.94%)	2009 (60.48%)	1756 (52.84%)	1,438 (43.27%)	
Yes	866 (26.06%)	1,313 (39.52%)	1,567 (47.16%)	1885 (56.73%)	
Hyperlipidemia (n, %)					<0.05
No	1804 (54.29%)	1,025 (30.85%)	441 (13.27%)	48 (1.44%)	
Yes	1,519 (45.71%)	2,297 (69.15%)	2,882 (86.73%)	3,275 (98.56%)	
CVD (n, %)					<0.05
No	3,118 (93.83%)	2,992 (90.07%)	2,904 (87.39%)	2,731 (82.18%)	
Yes	205 (6.17%)	330 (9.93%)	419 (12.61%)	592 (17.82%)	
HEI-2015	50.72 ± 13.37	50.63 ± 13.34	50.68 ± 13.14	50.43 ± 13.10	0.81
PhenoAge (years)	36.99 ± 19.51	45.96 ± 20.60	50.11 ± 20.19	55.33 ± 20.19	<0.05
PhenoAgeAccel (years)	−5.99 ± 6.72	−4.52 ± 7.63	−3.60 ± 7.47	−0.33 ± 10.27	<0.05

Abbreviations: TyG, triglyceride-glucose; PIR, poverty income ratio; MET, metabolic equivalent; BMI, body mass index; SBP, systolic blood pressure; DBP, diastolic blood pressure; eGFR, estimated glomerular filtration rate; CVD, cardiovascular disease; HEI, healthy eating index; PhenoAge, phenotypic age; PhenoAgeAccel, phenotypic age acceleration.

Metabolic and clinical parameters showed notable differences across TyG quartiles. BMI increased consistently with higher TyG levels, from 26.21 kg/m^2^ in Q1 to 30.80 kg/m^2^ in Q4 (P < 0.05). Blood pressure and uric acid levels also rose significantly across quartiles, as systolic blood pressure increased from 117.98 mmHg in Q1 to 129.76 mmHg in Q4, and uric acid levels rose from 4.94 mg/dL in Q1 to 5.97 mg/dL in Q4 (P < 0.05). Glucose metabolism state worsened, with the prevalence of diabetes increasing sharply from 4.30% in Q1 to 40.72% in Q4, while normoglycemia decreased from 89.41% in Q1 to 41.59% in Q4 (P < 0.05). Kidney function, as measured by eGFR, declined with higher TyG levels, dropping from 101.11 mL/min/1.73 m^2^ in Q1 to 87.56 mL/min/1.73 m^2^ in Q4 (P < 0.05). Age-related indicators such as PhenoAge and PhenoAgeAccel differed significantly, with PhenoAge rising from 36.99 years in Q1 to 55.33 years in Q4 and PhenoAgeAccel increasing from −5.99 years in Q1 to −0.33 years in Q4 (P < 0.05).

### 3.3 Association between TyG index and PhenoAgeAccel

The association between the TyG index and PhenoAgeAccel demonstrated a strong and significant positive relationship across all models, with adjustments progressively attenuating the effect size (Table 2). In the unadjusted model (Model 1), a one-unit increase in the continuous TyG index was associated with a 3.99-year increase in PhenoAgeAccel (95% CI: 3.79, 4.19). After adjusting for age, sex, and race/ethnicity (Model 2), this association slightly strengthened to 4.06 years (95% CI: 3.85, 4.27). In the fully adjusted model (Model 3), which accounted for a comprehensive set of covariates, the association remained statistically significant but was attenuated to 2.21 years (95% CI: 1.99, 2.43). Similarly, when analyzed by quartiles, participants in Q4 (highest TyG index) had significantly higher PhenoAgeAccel compared to Q1 (reference group), with an effect size of 5.66 years (95% CI: 5.27, 6.05) in Model 1, 5.51 years (95% CI: 5.10, 5.91) in Model 2, and 1.79 years (95% CI: 1.38, 2.20) in Model 3. A clear trend was observed across quartiles, with higher TyG quartiles associated with greater PhenoAgeAccel (P for trend <0.05 in all models).

**TABLE 2:** Association between TyG index and PhenoAgeAccel.

TyG index	Model 1	Model 2	Model 3
Continuous	3.99 (3.79, 4.19)	4.06 (3.85, 4.27)	2.21 (1.99, 2.43)
Quartiles			
Q1 (6.66–8.27)	Reference	Reference	Reference
Q2 (8.27–8.67)	1.48 (1.09, 1.87)	1.34 (0.95, 1.73)	0.52 (0.18, 0.86)
Q3 (8.67–9.11)	2.40 (2.00, 2.79)	2.30 (1.90, 2.70)	0.56 (0.19, 0.93)
Q4 (9.11–12.48)	5.66 (5.27, 6.05)	5.51 (5.10, 5.91)	1.79 (1.38, 2.20)
P for trend	<0.05	<0.05	<0.05

Model 1: Non-adjusted.

Model 2: Adjusted for age, sex, race/ethnicity.

Model 3: Adjusted for age, sex, race/ethnicity, PIR, education level, BMI, smoking, drinking, glucose metabolism state, hypertension, hyperlipidemia, METs/week, uric acid, eGFR, CVD, and HEI-2015.

Abbreviations: TyG, triglyceride-glucose; PhenoAgeAccel, phenotypic age acceleration; PIR, poverty income ratio; BMI, body mass index; MET, metabolic equivalent of task; eGFR, estimated glomerular filtration rate; CVD, cardiovascular disease; HEI, healthy eating index.

### 3.4 Model validation and diagnostic assessment

To ensure the validity and reliability of our findings, comprehensive model diagnostics and validation procedures were performed. Near-zero variance analysis confirmed that all variables included in the final models had sufficient variability to contribute meaningfully to the analysis. Multicollinearity assessment through VIF analysis revealed acceptable levels of correlation among predictors, with VIF values ranging from 1.1 to 3.1 for all variables in the final model, including TyG index (VIF = 1.7), BMI (VIF = 1.3), eGFR (VIF = 2.7), and uric acid (VIF = 1.6), indicating no problematic multicollinearity.

The fully adjusted model (Model 3) demonstrated good fit with an R^2^ of 0.3695 and adjusted R^2^ of 0.3682, indicating that approximately 37% of the variation in PhenoAgeAccel was explained by the model. Model comparison metrics included AIC (88200.8695) and log-likelihood (−44071.4347), with significant improvement in the null deviance from 936496.2415 to a residual deviance of 590506.7876, confirming the model’s explanatory power.

Residual analysis showed a standard deviation of 6.6658, and while the Pearson chi-square normality test (p < 0.0001) indicated some deviation from perfect normality, this is common in large datasets and does not substantially affect the robustness of our findings given the large sample size.

These comprehensive diagnostic assessments confirm the statistical validity and reliability of our findings regarding the association between TyG index and PhenoAgeAccel.

### 3.5 Threshold effect analysis of TyG index on PhenoAgeAccel

The threshold effect analysis revealed a non-linear relationship between the TyG index and PhenoAgeAccel, with a significant inflection point identified at 9.60 (Table 3). Using a standard linear model (Model I), the adjusted β for the association was 2.21 (95% CI: 1.99, 2.43, P < 0.05), suggesting a positive and linear association. However, Model II, which employed a piecewise linear regression, demonstrated distinct effects on either side of the inflection point. For TyG index values below 9.60, the adjusted β was 0.56 (95% CI: 0.29, 0.83, P < 0.05), indicating a weaker but statistically significant association. In contrast, for TyG values above 9.60, the association was substantially stronger, with an adjusted β of 8.21 (95% CI: 7.59, 8.82, P < 0.05). The log-likelihood ratio test supported the presence of a threshold effect (P = 0.02), highlighting a sharp increase in the impact of TyG index on PhenoAgeAccel beyond the inflection point.

**TABLE 3:** Threshold effect analysis of TyG index on PhenoAgeAccel.

TyG index	Adjusted β^*^ (95% CI)	P-value
Model I		
Fitting by the standard linear model	2.21 (1.99, 2.43)	<0.05
Model II		
Inflection point	9.60	
<9.60	0.56 (0.29, 0.83)	<0.05
>9.60	8.21 (7.59, 8.82)	<0.05
Log likelihood ratio	—	0.02

Notes: ^*^Adjusted for age, sex, race/ethnicity, PIR, education level, BMI, smoking, drinking, glucose metabolism state, hypertension, hyperlipidemia, METs/week, uric acid, eGFR, CVD, and HEI-2015.

Abbreviations: TyG, triglyceride-glucose; PhenoAgeAccel, phenotypic age acceleration; PIR, poverty income ratio; BMI, body mass index; MET, metabolic equivalent of task; eGFR, estimated glomerular filtration rate; CVD, cardiovascular disease; HEI, healthy eating index.

**FIGURE 2:**
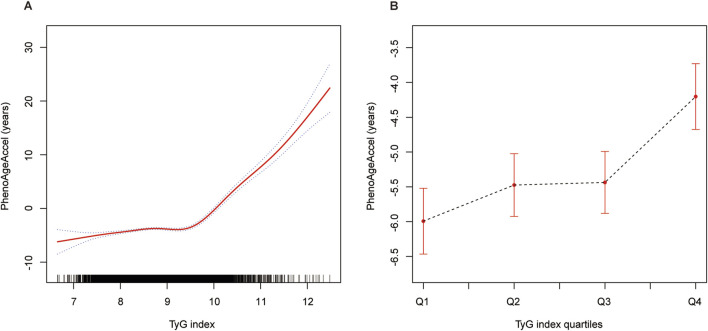
The association between TyG index and PhenoAgeAccel. Notes: **(A)** The nonlinear association between the TyG index and PhenoAgeAccel is depicted using GAM and smooth curve fitting. The solid red line represents the fitted curve, while the shaded area indicates the 95% confidence interval. **(B)** Smooth curve fitting of the TyG index after stratification into quartiles (Q1: 6.66–8.27, Q2: 8.27–8.67, Q3: 8.67–9.11, Q4: 9.11–12.48). The dashed black line represents the trend of PhenoAgeAccel across the quartiles, with error bars indicating the 95% confidence intervals. Age, sex, race/ethnicity, PIR, education level, BMI, smoking, drinking, glucose metabolism state, hypertension, hyperlipidemia, METs/week, uric acid, eGFR, CVD, and HEI-2015 were adjusted. Abbreviations: TyG, triglyceride-glucose; PhenoAgeAccel, phenotypic age acceleration; GAM, generalized additive model; PIR, poverty income ratio; BMI, body mass index; MET, metabolic equivalent of task; eGFR, estimated glomerular filtration rate; CVD, cardiovascular disease; HEI, healthy eating index.

The results presented in Figure 2 provide a visual representation of the threshold effect analysis described in Table 3. Panel A illustrates the association between the TyG index and PhenoAgeAccel as a continuous variable, showing a clear non-linear relationship with an inflection point identified at 9.6. Below this threshold, the association is relatively weak, while a steep increase in PhenoAgeAccel is observed for TyG index values above 9.6, consistent with the adjusted β estimates in Table 3 (0.56 below 9.6 and 8.21 above 9.6). Panel B further supports the presence of a threshold effect by presenting the smooth curve fitting after categorizing the TyG index into four quartiles. The plot demonstrates a progressively steeper increase in PhenoAgeAccel across quartiles, with the transition from Q3 to Q4 suggesting a potential inflection point in the relationship. This visualization aligns with the trends observed in Table 3 and underscores the non-linear association between TyG index and PhenoAgeAccel, particularly the heightened effect at higher TyG levels.

### 3.6 Stratified analysis of the association between TyG index and PhenoAgeAccel

**FIGURE 3:**
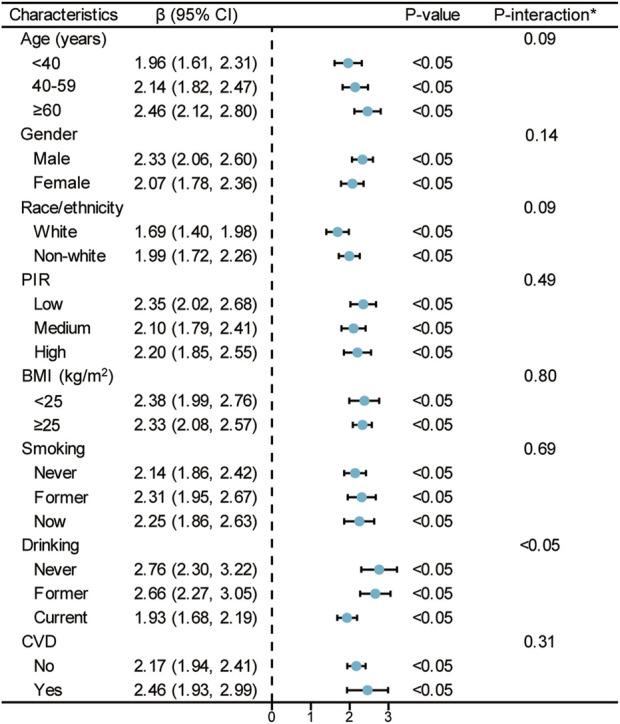
Stratified analyses between TyG index and PhenoAgeAccel. Notes: *Each stratification adjusted for all the factors (age, sex, race/ethnicity, PIR, education level, BMI, smoking, drinking, glucose metabolism state, hypertension, hyperlipidemia, METs/week, uric acid, eGFR, CVD, and HEI-2015) except the stratification factor itself. Abbreviations: TyG, triglyceride-glucose; PhenoAgeAccel, phenotypic age acceleration; PIR, poverty income ratio; BMI, body mass index; MET, metabolic equivalent of task; eGFR, estimated glomerular filtration rate; CVD, cardiovascular disease; HEI, healthy eating index.

Figure 3 depicts a subgroup analysis of the association between TyG index and PhenoAgeAccel using linear regression, stratified by age, gender, race/ethnicity, PIR, BMI, smoking, drinking, and CVD. The effect value (β) of TyG index on PhenoAgeAccel in all subgroups is greater than zero, with statistically significant P-values (P < 0.05) in all strata. This indicates that the TyG index maintains a consistent positive association with PhenoAgeAccel across all population subgroups. Notably, while the effect sizes vary slightly among subgroups, the relationship remains robust and significant, suggesting that the association between TyG index and accelerated phenotypic aging is not significantly modified by demographic or health-related factors.

**FIGURE 4:**
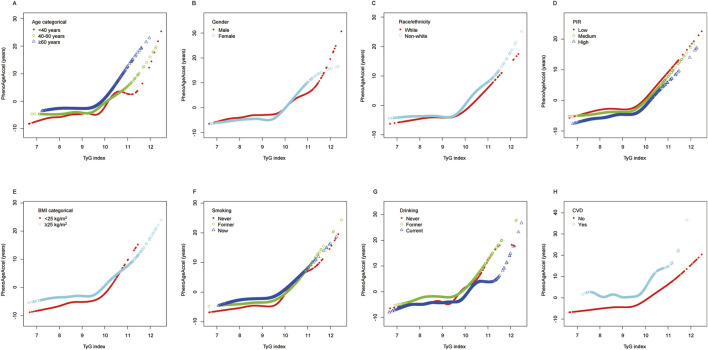
Stratified analyses (by **(A)** age; **(B)** gender; **(C)** race/ethnicity; **(D)** PIR; **(E)** BMI; **(F)** smoking; **(G)** drinking; **(H)** CVD) between TyG index and PhenoAgeAccel using GAM and smooth curve fittings. Notes: *Each GAM and smooth curve fitting was adjusted for all factors, including age, sex, race/ethnicity, PIR, education level, BMI, smoking, drinking, glucose metabolism state, hypertension, hyperlipidemia, METs/week, uric acid, eGFR, CVD, and HEI-2015, except for the stratification factor itself. Abbreviations: PIR, poverty income ratio; BMI, body mass index; CVD, cardiovascular disease; TyG, triglyceride-glucose; PhenoAgeAccel, phenotypic age acceleration; GAM, generalized additive model; MET, metabolic equivalent of task; eGFR, estimated glomerular filtration rate; HEI, healthy eating index.

Figure 4 illustrates the stratified analysis of the association between TyG index and PhenoAgeAccel using a generalized additive model and smooth curve fitting, stratified by the same variables as in Figure 3. The results are consistent with the trends observed in the total population analysis (Figure 2), demonstrating a potential non-linear relationship between TyG index and PhenoAgeAccel across all subgroups. For each stratified variable, the smooth curves generally exhibit an initial gradual increase in PhenoAgeAccel with lower TyG index values, followed by a more pronounced increase beyond higher TyG index levels, consistent with the non-linear threshold effect described earlier.

## 4 Discussion

This study investigated the association between the TyG index and PhenoAgeAccel using data from a nationally representative cohort. The results demonstrated a significant and positive relationship between the TyG index and PhenoAgeAccel, with higher TyG index values consistently associated with greater biological aging across all models. A non-linear threshold effect was identified, with an inflection point at a TyG index of 9.60; below this threshold, the association was modest, while above it, the effect size was substantially amplified. Stratified analyses revealed that the association persisted across all subgroups, including age, gender, race/ethnicity, PIR, BMI, smoking, drinking, and CVD, suggesting the robustness of the findings. These results highlight the critical role of metabolic dysfunction, as captured by the TyG index, in accelerating biological aging and emphasize the importance of addressing IR to mitigate aging-related health risks.

Our research findings are consistent with and extend previous studies exploring the relationship between metabolic dysfunction and biological aging. Numerous studies have demonstrated a close association between metabolic dysfunction and cellular aging. For example, one study found a significant negative correlation between various components of metabolic syndrome and leukocyte telomere length (LTL), suggesting that metabolic abnormalities may accelerate the process of cellular aging (Khalangot et al., 2019). Additionally, another study highlighted the cardiometabolic index (CMI) as a biomarker of biological aging, finding that elevated CMI levels are associated with an increased risk of biological aging (Sun and Bao, 2024). Similarly, Huang et al. demonstrated in a study involving patients with type 2 diabetes and coronary heart disease that higher levels of metabolic abnormalities significantly accelerate phenotypic aging (Huang et al., 2024). While Huang’s study focused on a specific population, our analysis utilized a nationally representative NHANES sample (n = 13,291), providing broader generalizability. Likewise, Zhang et al. showed that higher TyG index levels are associated with lower levels of the anti-aging protein Klotho, further supporting the relationship between metabolic dysfunction and aging (Zhang et al., 2024). However, Zhang’s study had a smaller sample size (n = 2,864) and did not explore nonlinear associations. Our study employed GAM and segmented regression to identify threshold effects.

In addition, this study found a significant nonlinear relationship between the TyG index and PhenoAgeAccel, with a turning point observed when the TyG index reached 9.60. This finding suggests that the impact of an elevated TyG index on biological aging is not simply linear but exhibits a clear threshold effect. Below the turning point, the association between the TyG index and PhenoAgeAccel is relatively weak, whereas above the turning point, this relationship becomes significantly stronger. The discovery of this nonlinear relationship provides a new perspective on how metabolic disorders drive biological aging and may hold important implications for risk stratification and personalized intervention strategies. This result aligns with findings from other studies that have identified nonlinear effects of metabolic markers on health outcomes. For example, Qiu et al. investigated the relationship between the TyG index and the anti-aging protein Klotho, also observing that the negative health impacts of metabolic indicators significantly increased beyond specific thresholds (Qiu et al., 2024). Furthermore, Xu et al. found that the relationship between the visceral adiposity index (VAI) and biological aging was significantly enhanced after certain critical points, consistent with the threshold effect observed in this study (Xu et al., 2024). However, unlike these studies, the present research more precisely quantified this nonlinear relationship using GAM and segmented regression methods, and for the first time, identified the exact location of the turning point in a nationally representative sample.

This nonlinear relationship may be associated with the cascade effects of IR, oxidative stress, and chronic inflammation. When the TyG index is relatively low, metabolic disturbances are insufficient to cause significant damage to cells and tissues, resulting in a weaker impact on PhenoAgeAccel. However, once the TyG index exceeds the threshold of 9.60, IR may intensify, triggering pro-inflammatory pathways (such as NF-κB and JNK) and oxidative stress, leading to DNA damage, mitochondrial dysfunction, and an escalation of inflammatory responses (Qiu et al., 2024; Zhao et al., 2024). The identification of this specific threshold at TyG index of 9.60 may reflect critical transitions in metabolic pathophysiology. This finding aligns with previous research by Primo et al., who identified specific TyG index cutoff points as accurate markers for predicting metabolic syndrome in obese subjects, suggesting that metabolic parameters exhibit threshold effects rather than continuous linear relationships with health outcomes (Primo et al., 2023). The TyG index has been increasingly recognized as a promising biomarker for various diseases, with different threshold values indicating distinct risk profiles for different conditions, as reported by Sun et al. in their comprehensive review (Sun et al., 2025). The mechanisms underlying this threshold effect likely involve multiple interconnected pathways. First, the transition from compensated to decompensated metabolic function may occur around this threshold. The TyG index is strongly associated with insulin resistance, which at higher levels promotes a pro-inflammatory phenotype characterized by increased secretion of pro-inflammatory cytokines and chemokines. This phenomenon, often referred to as “inflammaging,” represents a chronic low-grade inflammation that is a hallmark of accelerated biological aging, as described by Bachmann et al. and Zuo et al. in their reviews of inflammatory mechanisms in aging (Bachmann et al., 2020; Zuo et al., 2019). Second, oxidative stress increases exponentially rather than linearly with worsening metabolic health. Lejawa et al. demonstrated that individuals with unhealthy metabolic phenotypes exhibit significantly higher levels of oxidative stress markers and shorter telomere length compared to metabolically healthy counterparts (Lejawa et al., 2021). This oxidative damage to cellular components, including lipids, proteins, and DNA, contributes substantially to the aging process and the development of age-related diseases, as highlighted by Griffiths et al. in their analysis of redox state dysregulation in aging (Griffiths et al., 2020). Third, epigenetic modifications likely play a crucial role in mediating the effects of metabolic dysfunction on biological aging. The TyG index may affect epigenetic aging by altering the expression of genes involved in metabolic pathways, inflammation, and oxidative stress. Cao et al. have described how mitochondrial epigenetic changes (“mitoepigenetics”) serve as an important regulatory layer in aging and metabolic-related diseases (Cao et al., 2021). Additionally, Wolf et al. found significant correlations between epigenetic age acceleration and altered gene expression profiles, suggesting that metabolic factors could influence aging through epigenetic mechanisms (Wolf et al., 2021). Furthermore, IR may inhibit DNA repair pathways and alter epigenetic markers, thereby permanently activating aging-related genes (Park et al., 2014). Disruptions in endocrine balance, such as reduced levels of Klotho protein, may also amplify this effect (Zhang et al., 2024). Therefore, an elevated TyG index may promote biological aging through multiple pathways, with more pronounced effects observed beyond the critical threshold.

The molecular mechanisms connecting TyG index with PhenoAgeAccel extend beyond the previously discussed pathways. Recent studies have suggested that the TyG index is associated with epigenetic age acceleration, which is a measure of biological aging based on DNA methylation patterns (Irvin et al., 2018). This association provides a direct molecular link between metabolic dysfunction and aging processes at the genomic level. Furthermore, the TyG index has been linked to the anti-aging protein Klotho, which plays a crucial role in cellular longevity and metabolic health (Qiu et al., 2024). Studies have found that the relationship between the TyG index and Klotho levels varies depending on the presence of diabetes, indicating a complex interaction that may contribute to differential aging outcomes in various metabolic states. Additionally, the TyG index has demonstrated utility in predicting liver steatosis, a condition associated with metabolic syndrome that may contribute to accelerated aging through disruption of normal liver function and metabolism (Mijangos-Trejo et al., 2024). The potential connections between the TyG index and these molecular markers suggest multiple pathways through which metabolic dysfunction might influence biological aging, beyond the previously discussed inflammation and oxidative stress mechanisms. These findings align with broader research on metabolic factors and aging, including studies on genetic determinants of aging and metabolic health (Ruth et al., 2021; Chen et al., 2023). Further research is needed to fully elucidate these mechanistic connections and identify potential intervention targets to mitigate accelerated aging associated with metabolic disorders.

The stratified analysis results of this study indicate that, across different stratification variables, although the effect size (β value) showed slight variations, the association between the TyG index and PhenoAgeAccel remained significant in all subgroups, demonstrating its consistency across various populations. This finding aligns with previous research, such as studies by Qiu et al. and Zhang et al., which similarly observed that the relationship between metabolic markers and aging remains consistent across the majority of population subgroups (Qiu et al., 2024; Zhang et al., 2024). Notably, in the interaction effect test, the P-value for drinking status was less than 0.05, suggesting a certain interaction effect between alcohol consumption and the relationship between the TyG index and PhenoAgeAccel. Stratified analysis revealed that the association effect among drinkers (particularly current drinkers) was slightly lower than that of never drinkers and former drinkers, which may be related to the bidirectional effects of alcohol consumption on metabolism and inflammatory responses. Makino et al. demonstrated that light to moderate alcohol consumption was positively correlated with favorable aging-related markers, including improved lung function and muscle mass, potentially offering some protective effects against certain aspects of biological aging (Makino et al., 2021). However, the relationship becomes more complex when considering the TyG index, which is a marker of insulin resistance. Li et al. found that high TyG index values are associated with increased risk of cognitive decline in middle-aged to elderly populations, highlighting its role in aging processes (Li et al., 2022). Further complicating this relationship, Keskin and Yoldas reported that fructose consumption, which is often high in alcoholic beverages, correlates with the TyG index and negatively affects glycemic status, potentially exacerbating metabolic dysfunction (Keskin and Yoldas Ilktac, 2022). The underlying mechanisms for this interaction may involve several pathways. First, moderate alcohol consumption may influence insulin sensitivity and glucose metabolism through hormonal and inflammatory mediators, potentially modifying the relationship between TyG index and biological aging (Sánchez-Ortiz et al., 2024; Alonso-Andrés et al., 2019). Second, alcohol’s effects on oxidative stress are dose-dependent, with moderate consumption potentially enhancing antioxidant capacity while excessive intake promotes oxidative damage, as demonstrated by Hong’s research on oxidative stress and plasma alcoholic metabolites (Hong, 2016). Third, the lifestyle factors often associated with different patterns of alcohol consumption, such as dietary habits and physical activity, may confound or mediate the relationship between metabolic health and aging. Hautekiet et al. highlighted that a healthy lifestyle, which may include moderate alcohol consumption within an overall balanced approach, is associated with favorable biological aging markers such as telomere length and mitochondrial DNA content (Hautekiet et al., 2022). These complex interactions underscore the importance of considering alcohol consumption as a potentially significant modifier in the relationship between metabolic dysfunction and biological aging. Future research should explore these interactions more comprehensively, particularly focusing on the dose-dependent effects of alcohol consumption on the relationship between TyG index and biological aging, and the potential mechanisms underlying the observed modification effect.

A notable strength of this study lies in its comprehensive methodology, which integrates advanced statistical techniques to explore the relationship between the TyG index and PhenoAgeAccel. The use of GAM and smooth curve fitting enabled the identification of a non-linear association, highlighting the inflection point at a TyG index of 9.60. This approach provides a nuanced understanding of threshold effects that traditional linear regression models may overlook (Hastie and Tibshirani, 1987). Furthermore, the application of MICE minimized bias from missing data and ensured robust findings, a method endorsed in epidemiological studies (Toutenburg and Rubin, 1990; Sterne et al., 2009). Additionally, the detailed stratified analysis across demographic and clinical subgroups demonstrated consistent associations, underscoring the universal relevance of the TyG index as a biomarker of biological aging.

While this study provides valuable insights, several limitations should be acknowledged. First, the cross-sectional design prevents the establishment of causality, leaving uncertainty about whether higher TyG index levels directly accelerate biological aging or are merely associated with it. Second, the reliance on self-reported data for covariates such as smoking and alcohol use may introduce recall bias (Hansen et al., 2022). Third, although we adjusted for multiple covariates, residual confounding from unmeasured factors cannot be ruled out. Specifically, our study was limited in its ability to account for detailed dietary patterns beyond the HEI-2015 score, objectively measured physical activity, sleep quality, and genetic predispositions, all of which may influence both metabolic health and biological aging processes. Future studies should aim to integrate more comprehensive data on diet composition, objectively measured physical activity using accelerometers, sleep metrics, and relevant genetic polymorphisms to better understand the complex relationship between metabolic dysfunction and biological aging. Finally, the generalizability of our findings is limited by the characteristics of the NHANES population. While NHANES is designed to be representative of the U.S. population, our results may not be applicable to populations from other geographic regions with different genetic backgrounds, dietary habits, environmental exposures, and healthcare systems. Future research should prioritize including racially and ethnically diverse cohorts from various geographic regions to establish the consistency of the relationship between TyG index and biological aging across different populations and to identify potential population-specific modifying factors.

## 5 Conclusion

This study underscores the relevance of metabolic health, particularly as captured by the TyG index, in understanding biological aging and its potential impact on health outcomes. The TyG index serves as a practical and accessible marker of metabolic dysfunction, which plays a critical role in aging-related processes. Early detection of elevated TyG index, especially values approaching or exceeding the identified threshold of 9.60, offers opportunities for timely intervention to mitigate accelerated biological aging.

Potential interventions include targeted lifestyle modifications, such as Mediterranean or DASH dietary patterns that improve triglyceride and glucose profiles, structured physical activity programs combining aerobic and resistance training, and adequate sleep hygiene—all of which have demonstrated efficacy in improving insulin sensitivity and lowering TyG index values. Pharmacological approaches may also prove beneficial, including metformin, which has shown promise in addressing both metabolic dysfunction and aging processes, as well as SGLT2 inhibitors and GLP-1 receptor agonists that improve glycemic control and cardiovascular outcomes.

Future research should focus on investigating how changes in metabolic markers over time influence aging trajectories. Expanding these investigations to include diverse populations with varying genetic, environmental, and lifestyle factors will improve the generalizability of findings. Furthermore, interventional studies targeting improvements in TyG index through lifestyle modifications or pharmacological approaches could provide more definitive evidence regarding the potential benefits of metabolic optimization on biological aging processes.

Integrating molecular and epigenetic biomarkers with metabolic indices could provide a more comprehensive understanding of the mechanisms driving biological aging. These efforts will be essential for developing personalized strategies aimed at enhancing healthy aging and reducing the burden of age-related diseases.

## Data Availability

The raw data supporting the conclusions of this article will be made available by the authors, without undue reservation.
